# Membranes are functionalized by a proteolipid code

**DOI:** 10.1186/s12915-024-01849-6

**Published:** 2024-02-27

**Authors:** Troy A. Kervin, Michael Overduin

**Affiliations:** 1 Division of Structural Biology, Wellcome Trust Centre for Human Genetics, University of Oxford, Oxford, OX3 7BN UK; 2 Department of Biochemistry, University of Alberta, Edmonton, AB Canada

**Keywords:** Fingerprint, Integral membrane protein, Lipidon, Peripheral membrane protein, Phosphoinositide, Protein island, Proteolipid code, Zone, Zoning

## Abstract

Membranes are protein and lipid structures that surround cells and other biological compartments. We present a conceptual model wherein all membranes are organized into structural and functional zones. The assembly of zones such as receptor clusters, protein-coated pits, lamellipodia, cell junctions, and membrane fusion sites is explained to occur through a protein-lipid code. This challenges the theory that lipids sort proteins after forming stable membrane subregions independently of proteins.

## Membrane zonation

Membranes are organized fluid structures that incorporate many different proteins and lipids. Their basic architecture consists of a lipid bilayer with embedded integral proteins and peripheral proteins that associate with a single leaflet. While more than 50 years have passed since this architecture was revealed [1], there is no consensus on how proteins and lipids interact to create distinct functional regions within the same continuous fluid. To resolve this quandary, it is useful to refer to any of the unique membrane regions with a single term so that explanations can be generalized, although “membrane raft” refers only to regions enriched in sterols and sphingolipids [2], while “domain” ambiguates with protein domains. Here, we formulate an explicit framework for membrane structure centred around the concept of a “zone” [3–5], which we define as a region of membrane enclosing a contiguous group of particles capable of working together to perform a biological function.

In our endeavour to comprehensively describe membrane structure, we explain what zones are and how they are created and regulated. Examples of zones include clathrin-coated pits, microvilli, lamellipodia, cholesterol- and sphingolipid-enriched regions [2, 6], neuronal synaptic termini, cell junctions, membrane contact sites, tetraspanin-enriched “microdomains”, bacterial “microdomains” [7], receptor clusters, viral budding sites [8], and integral proteins stabilized by lipid “fingerprints” [9–11]. These functional units are created by a protein-lipid language that harmonizes with the genetic code to direct the flow of information in cells; hence, we call our model the proteolipid code.

## Hierarchy of zone attributes

**Fig. 1:**
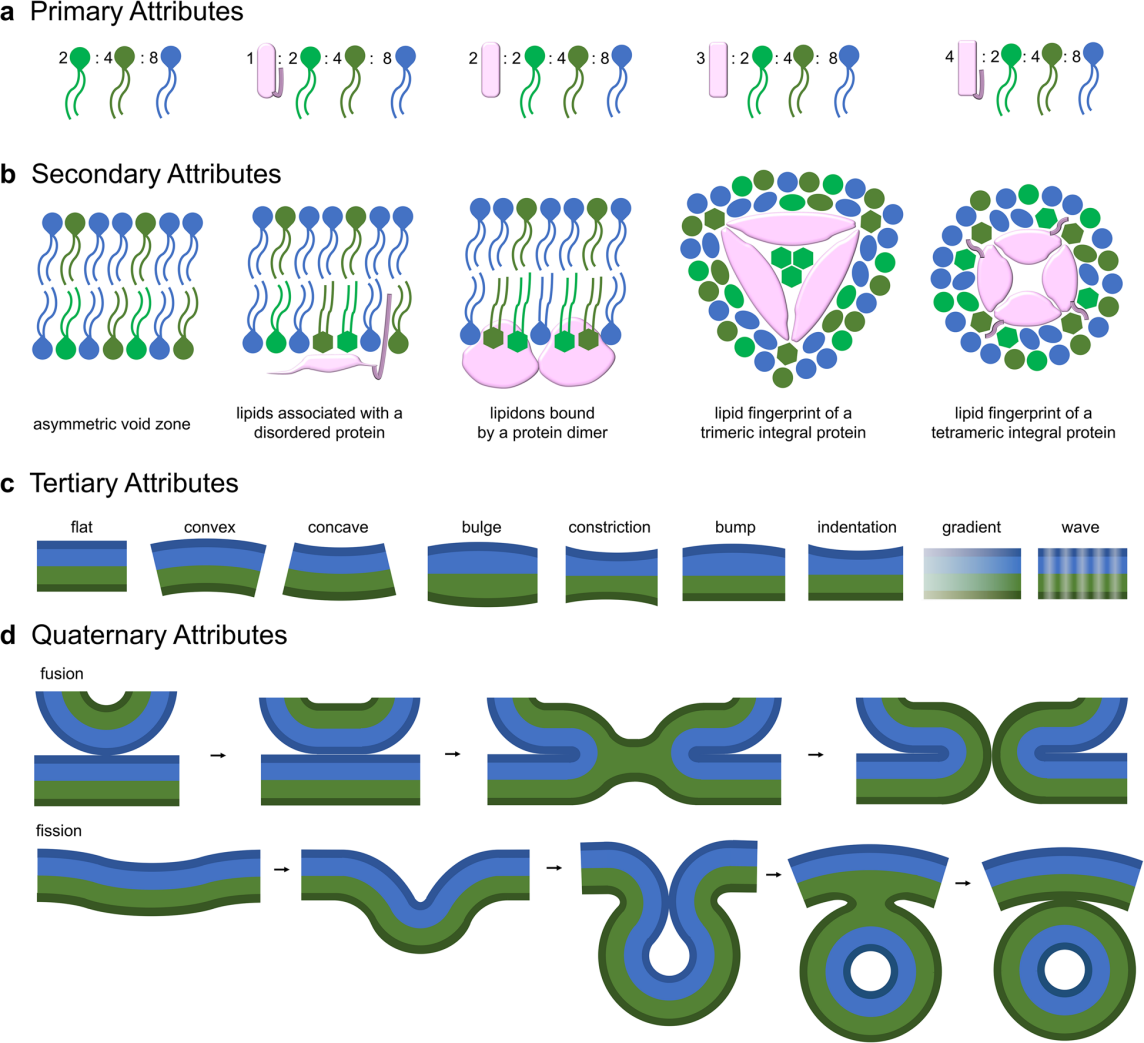
Hierarchy of zone attributes. **a** Zone primary structures with proteins as pink shapes and lipids as circles with two tails or one tail attached to protein. **b** Secondary structures in either side or top views with proteins in pink and lipids as circles, ovals, or hexagons representing free, loosely bound, and strongly bound to protein, respectively. **c** Tertiary attributes illustrated on a bilayer with one leaflet coloured blue and the other green. **d** Quaternary attributes illustrated by merging and separating zones

**Fig. 2:**
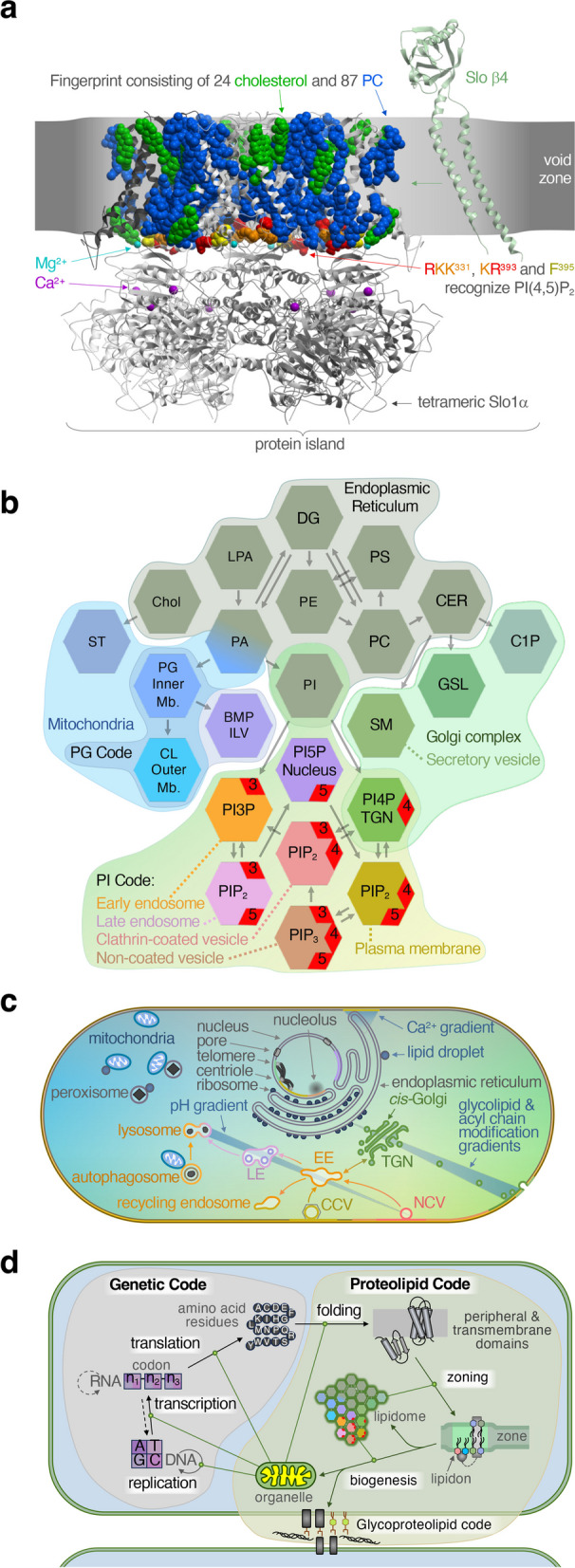
Representations of the proteolipid code. **a** Structure of a zone resolved by cryogenic electron microscopy containing the Slo1 channel α tetramer [14] (PDB ID: 8GHF, silver ribbons) and interacting β4 subunit [15] (PDB ID: 6V22, light green). The channel has fingerprints of cholesterol (green) and PC (blue) [14], recognizes PI(4,5)P_2_ via basic (orange and red) and aromatic (yellow) residues [16–18], and is regulated by Ca^2+^ (magenta spheres) and coordinates Mg^2+^ (aqua). Created with ICM-browser. For a model to be called a zone, it should contain a set of molecules capable of performing a biological function in a membrane. **b** The lipidome depicted with hexagons representing individual molecules with their standard IUPAC abbreviations and organized by their major but nonexclusive subcellular origin [19, 20]. PIP terminal phosphate numbers are displayed on red corners and arrows indicate enzymatic reactions that convert these lipids. **c** Eukaryotic organelles are depicted and coloured as in **b**. Lipid aliphatic chains have different lengths and degrees of unsaturation which vary by zone. **d** The biological code composed of the genetic and proteolipid codes

We will first explain how zones can be conceptualized with analogy to the structure hierarchy that describes proteins (Fig. 1). A hierarchy for membrane structure has already been proposed [12, 13]; however, it is more convenient when defined in relation to zones. The primary structure of a zone is its lipid and protein composition, which can be determined with methods such as mass spectrometry. Secondary structure is the spatial arrangement of zone components, including bilayer asymmetry and non-random lateral distributions. This can be probed by extracting physiological membranes and visualizing them with a structural technique such as cryogenic election microscopy (Fig. 2a) [14]. Tertiary features are supramolecular properties such as curvature, thickness, fluidity, density, electrostatic charge, and gradient or wave characteristics and can be observed in molecular simulations [12]. Finally, quaternary relationships are interactions between zones such as the exchange of molecules and the intake or loss of membrane sections. These can be seen with techniques such as single particle tracking or by imaging formations like budding vesicles, elongating tubules, membrane contact sites between organelles, or hemi-fusion intermediates.

All four zone attribute ranks have functional significance. For instance, secondary structure is vital for integral proteins such as receptors and ion channels, which require bound lipids for stability and sometimes regulation [9, 21]. Furthermore, colocalized molecules belonging to secondary structure can be coincidentally detected by proteins that are recruited from the cytosol [22, 23]. Tertiary features such as curvature create the morphology of organelles and can be sensed by crescent-shaped BAR domains [24]. Stiffness is also an important tertiary characteristic that plays a role in vesicle genesis and viral fusion [25, 26]. Quaternary associations are the materialization of direct communication between membranous organelles, cells, and viruses. Secondary, tertiary, and quaternary attributes each originate from primary structure, which is organized by integral and peripheral proteins.

## Zoning by integral proteins

We begin our discussion on zoning by summarizing the role of integral proteins, which account for about half the mass of physiological membranes [27, 28] and approximately 26% of the human proteome [29]. Integral proteins possess unique lipid “fingerprints” by virtue of their preferential interactions [10]. They may also have strong close-range interactions with each other, directly or through their fingerprints [30], which can cause them to cluster into “islands” [21, 31, 32], particularly when lipids are scarce, when hydrophobic domains have matching lengths, or when there is mismatch between hydrophobic domains and lipid aliphatic chains [33]. When integral proteins cluster, they leave surplus lipids not occupying their remaining fingerprint sites to pool into adjacent “void” zones [4, 31]. Consequently, integral proteins form lipid fingerprints, islands, and lipid-only voids.

This theory of zoning, which also extends to membrane-anchored peripheral proteins, explains observations that protein-independent lipid clustering does not. The proportion of Ras proteins found in cholesterol-rich zones is independent of protein expression level [34, 35], suggesting that proteins recruit lipids that then locally adopt ordered states [36]. Several distinct zones that are each enriched in a particular type of protein may coexist, such as those containing receptors and adaptors in T cells [37]. This indicates that compatible proteins induce their own zones rather than being recruited to preexisting ones [21, 36]. Protein clustering is the simplest explanation for protein-rich and lipid-only zones as well as the observation that not all of the former qualify as “rafts” [31]. Overall, the idea of integral protein recruitment to self-assembling lipid subregions should be rejected in favour of our proposal of zoning through the proteolipid code.

## Zone recognition by peripheral proteins

Before describing how peripheral proteins create or remodel zones, it is useful to explain one way that they are recruited to zones in the first place. Many eukaryotic proteins bind phosphatidylinositol phosphates (PIPs) [23], which, like other lipids, are enriched or depleted in specific zones in each organelle membrane (Fig. 2b, c). For example, the early endosome (EE), trans-Golgi network (TGN), and plasma membranes each respectively have PI3P, PI4P, and PI(4,5)P_2_ as signature lipids. We propose that proximal sets of lipids, especially including PIPs, act as “lipid codons” or “lipidons” that represent zone-specific identities recognized by peripheral domains [22, 23]. Recognition of lipidons, sometimes with coinciding signals such as protein motifs, pH, or metal ions, explains how proteins with weak affinity for solitary molecules attach to membranes with sufficient strength [22]. Crystal structures [38–42] and molecular simulations [43–45] show in detail how multiple PIPs and other lipids reside in adjacent pockets in representative peripheral domains.

## Zoning by peripheral proteins

As peripheral proteins bind lipidons, they will simultaneously manipulate the zone that they attach to [46, 47]. This is exemplified by the binding of immature human immunodeficiency virus Gag to the plasma membrane inner leaflet. The Gag polyprotein targets the plasma membrane by recognizing lipids via its matrix domain [48, 49], inducing large-scale sequestration of PI(4,5)P_2_ as well as cholesterol while multimerizing to create a zone that cooperatively recruits more Gag monomers [8]. This flexes the plasma membrane until the nascent virion buds, where the enrichment of PI(4,5)P_2_ and cholesterol in the viral membrane signifies its origin from an induced zone [50]. The recognition of lipidons on protein islands can also create tubules and protein-coated pits [51–53]. To generalize, any force exerted on the membrane can induce, remodel, or maintain a zone [47], including intercellular, cell wall, and cytoskeleton interactions.

## The cytoskeleton

The actin-based cytoskeleton fixes to protein islands and is important for their stability [31]. It also induces zones such as lamellipodia, filopodia, podosomes, and invadosomes by recognizing lipidons with actin adaptors [54, 55]. Kusumi et al. propound that the cytoskeleton immobilizes rows of integral proteins that maintain 40–300-nm compartments on the plasma membrane through steric hindrance and hydrodynamic friction within the bilayer [13]. These compartments supposedly contain all other zones such as “rafts”. We disagree with this characterization and believe that these “rafts” and their delimiting “compartments” are one and the same; that is, they are protein islands attached to the cytoskeleton.

## Regulation of zoning

Our theories imply that zoning is affected by membrane protein expression, degradation, and activity including enzymatic reactions that produce, convert, and degrade lipids. Enzymatic reactions may also create momentary void zones that are depleted in reactant or enriched in product, which can manifest as a molecular wave [56]. For peripheral proteins, post-translational modifications on lipidon attachment surfaces are positioned to impede or enhance zone recognition. For instance, phosphorylation and lysine modifications appear to reduce net positive charges and disrupt hydrogen bonds to segregate proteins from acidic lipids [57–59]. Conversely, covalent modification with lipids such as fatty acids, isoprenoids, and glycosylphosphatidylinositol can act as covalently attached lipidon components [21]. For example, farnesyl and geranylgeranyl groups may match zones with smaller and larger hydrophobic thicknesses, respectively. This would explain the diversity of lipid types that are attached to proteins.

## Conclusions

It has been remarked that a single, comprehensive model of the membrane is no longer attainable [60]. This sentiment reflects an incohesive research programme where many hypotheses are available and inaccurate ones are not abandoned [13, 61, 62]. Among those that should be abandoned, the theory that proteins sort to lipid subregions that form independently of proteins [6, 63] is foremost. That said, we do not mean to devalue lipid-lipid interactions, which maintain the membrane bilayer and influence zone attributes. Additionally, zone attributes can be used to condense the many hypotheses regarding the membrane localization of peripheral proteins. We arrive at a model that incorporates the following principles:

- Membranes are divided into zones which are their structural and functional building blocks and which are characterized by their primary, secondary, tertiary, and quaternary attributes.
- Zoning has synchronous protein and lipid dependency; therefore, the instructions for membrane function are specified in a proteolipid code.
- Integral proteins give rise to three zone types: integral proteins together with lipid fingerprints, protein islands, and lipid-only voids.
- Peripheral proteins and other membrane-binding objects modify zones by recognizing primary, secondary, and tertiary zone attributes.
- Zoning is regulated by changes in protein and lipid concentration or state, such as by enzymatic reactions.
- The proteolipid code is interdependent with the genetic code, and together, they direct the flow of molecular information in cells.

Further to our final point, the proteolipid code completes the cycle of information that allows cells to function (Fig. 2d). This “biological code” can be understood to begin with DNA, which contains the information needed to make RNA and the amino acid sequences of proteins. Proteins create and interact with other biomolecules to conduct most of the business of life, including synthesizing and engaging lipids to create membranes. Membranes are the apotheosis of the biological code as they enclose the entire cell and play a role in nearly all processes, including modulating transcription [64] and organizing ribosomal arrays [65]. The flow of information ends with the degradation of molecules, for instance, at proteasomes which cluster at the endoplasmic reticulum [66]. This cycle equips us to fathom the continuity of life.

## Data Availability

Not applicable.

## Abbreviations

BMP: Bis(monoacylglycerol)phosphate
CL: Cardiolipin
CER: Ceramide
C1P: Ceramide-1-phosphate
Chol: Cholesterol
CCV: Clathrin-coated vesicle
DG: Diacylglycerol
EE: Early endosome
GSL: Glycosphingolipid
ILV: Intralumenal vesicle
IUPAC: International Union of Pure and Applied Chemistry
LE: Late endosome
LPA: Lysophosphatidic acid
NCV: Non-coated vesicle
PA: Phosphatidic acid
PC: Phosphatidylcholine
PE: Phosphatidylethanolamine
PI: Phosphatidylinositol
PIP: Phosphatidylinositol phosphate
PI3P: Phosphatidylinositol 3-phosphate
PI4P: Phosphatidylinositol 4-phosphate
PI5P: Phosphatidylinositol 5-phosphate
PIP_2_: Phosphatidylinositol bisphosphate
PI(4,5)P_2_: Phosphatidylinositol 4,5-bisphosphate
PIP_3_: Phosphatidylinositol trisphosphate
PG: Phosphatidylglycerol
PS: Phosphatidylserine
PDB: Protein Data Bank
SM: Sphingomyelin
ST: Sterol
TGN: Trans-Golgi network
